# Multiparametric Skin Assessment in a Monocentric Cohort of Systemic Sclerosis Patients: Is There a Role for Ultra-High Frequency Ultrasound?

**DOI:** 10.3390/diagnostics13081495

**Published:** 2023-04-21

**Authors:** Marco Di Battista, Simone Barsotti, Saverio Vitali, Marco Palma, Giammarco Granieri, Teresa Oranges, Giacomo Aringhieri, Valentina Dini, Alessandra Della Rossa, Emanuele Neri, Marco Romanelli, Marta Mosca

**Affiliations:** 1 Rheumatology Unit, University of Pisa, 56124 Pisa, Italy; 2 Department of Medical Biotechnologies, University of Siena, 53100 Siena, Italy; 3 Radiology Unit, University of Pisa, 56124 Pisa, Italy; 4 Dermatology Unit, University of Pisa, 56124 Pisa, Italy

**Keywords:** systemic sclerosis, ultra-high frequency ultrasound, durometry, skin score, skin imaging, skin fibrosis

## Abstract

*Background*: To assess skin involvement in a cohort of patients with systemic sclerosis (SSc) by comparing results obtained from modified Rodnan skin score (mRSS), durometry and ultra-high frequency ultrasound (UHFUS). *Methods*: SSc patients were enrolled along with healthy controls (HC), assessing disease-specific characteristics. Five regions of interest were investigated in the non-dominant upper limb. Each patient underwent a rheumatological evaluation of the mRSS, dermatological measurement with a durometer, and radiological UHFUS assessment with a 70 MHz probe calculating the mean grayscale value (MGV). *Results*: Forty-seven SSc patients (87.2% female, mean age 56.4 years) and 15 HC comparable for age and sex were enrolled. Durometry showed a positive correlation with mRSS in most regions of interest (*p* = 0.025, ρ = 0.34 in mean). When performing UHFUS, SSc patients had a significantly thicker epidermal layer (*p* < 0.001) and lower epidermal MGV (*p* = 0.01) than HC in almost all the different regions of interest. Lower values of dermal MGV were found at the distal and intermediate phalanx (*p* < 0.01). No relationships were found between UHFUS results either with mRSS or durometry. *Conclusions*: UHFUS is an emergent tool for skin assessment in SSc, showing significant alterations concerning skin thickness and echogenicity when compared with HC. The lack of correlations between UHFUS and both mRSS and durometry suggests that these are not equivalent techniques but may represent complementary methods for a full non-invasive skin evaluation in SSc.

## 1. Introduction

Systemic sclerosis (SSc) is a rare connective tissue disorder characterized by diffuse microangiopathy and immune dysregulation, pathogenic elements that ultimately lead to the most known feature of this disease, namely tissue fibrosis of skin and internal organs [1,2]. Skin thickening is one of the most evident and studied aspects of SSc since it can be easily analyzed and mostly because it has been widely demonstrated that a more extensive skin involvement correlates with more severe internal organ damage, poor prognosis and increased disability [3,4]. Modified Rodnan Skin Score (mRSS) is a manual semi-quantitative score representing the most widespread clinometric tool to assess skin thickening in SSc. Despite the possible intra-reader and especially inter-reader variability, it is used as a primary or secondary outcome measure in clinical trials [5].

To improve the accuracy and sensitivity to change in the measurement of skin involvement, the use of semiquantitative/quantitative methods has been proposed. Among them, the durometer proved to be a non-invasive and easy-to-use instrument that can provide accurate and reliable measurements, gaining consideration as a complementary method to mRSS [6]. Recently, cutaneous ultrasonography emerged as a remarkable technique which allows for quantifying skin thickness, and studies regarding its use in SSc skin assessment have been published so far [7]. In the last few years, a growing interest has arisen in ultra-high frequency ultrasound (UHFUS), a method allowing a non-invasive detailed characterization of skin layers [8].

Our work aims to assess cutaneous involvement in a cohort of SSc patients, comparing results obtained from mRSS, durometry and UHFUS to obtain a complete and accurate multiparametric non-invasive evaluation of SSc skin.

## 2. Materials and Methods

### 2.1. Patients

We enrolled adult patients fulfilling 2013 EULAR/ACR criteria for SSc [9] attending a routine outpatient visit at the Rheumatology Unit of the University of Pisa between January and December 2019. We also enrolled 15 healthy controls (HC) with the same mean age and sex percentage of the SSc cohort. Full ethical approval was obtained from the local ethical committee (Comitato Etico Area Vasta Nord Ovest, approval number 13408). Each subject voluntarily agreed to participate and gave written informed consent to publish the material.

Each patient underwent a multidisciplinary (rheumatological, dermatological and radiological) evaluation at enrolment time. Initial data were collected through questions, medical records, and physical examination, including epidemiological data, disease duration and autoantibody profile (distinguishing between anti-centromere—ACA and anti-topoisomerase I—Scl70). All ongoing therapies were allowed. According to LeRoy classification [10], patients were classified into three skin subsets: sine scleroderma (ssSSc), limited (lcSSc) and diffuse cutaneous (dcSSc) groups. Moreover, the presence of hand contractures and the history of digital ulcers (DUs) were investigated. Ongoing DUs were an exclusion criterion. Afterwards, mRSS was performed either in the whole body (0–51 pts) or in some specific regions of interest (0–3 pts each) of the non-dominant upper limb:

- on the central dorsal side of the intermediate phalanx (IP) of the second finger.
- on the central dorsal side of the proximal phalanx (PP) of the second finger.
- on the dorsum of the hand (DH) (3 cm distally to the wrist joint).
- on the volar side of the forearm (VF) (5 cm proximally to the wrist joint).

### 2.2. Instrumental Assessment

Durometry assessment was performed by a single operator on five regions of interest of the non-dominant upper limb: distal phalanx (DP), IP and PP of the second finger, DH and VF. Skin hardness was measured with a portable durometer (Rex Model 1600, Rex Gauge Company, Buffalo Grove, IL, USA) in standard durometer units. The durometer was held in a vertical position keeping the foot of the gauge firmly against the skin. Each area was assessed three times, considering the mean value as the result.

**Figure 1:**
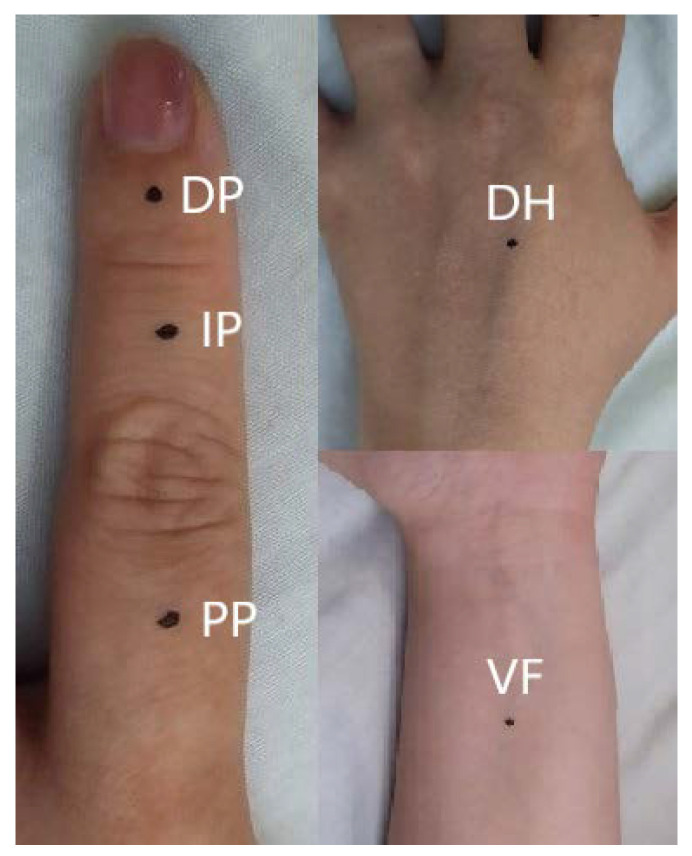
Skin regions of interest undergoing multiparametric non-invasive assessment. DP: distal phalanx; IP: intermediate phalanx; PP: proximal phalanx; DH: dorsum hand; VF: volar forearm.

UHFUS assessment was performed by a single operator with a 70 MHz probe (Vevo MD, VisualSonics, Toronto, ON, Canada). Images were acquired by placing a homogeneous layer of ultrasound gel between the transducer and the skin, keeping the various acquisition parameters constant. Five-second static clips of the skin were acquired at the five sites mentioned above DP, IP, PP, DH, and VF (Figure 1). A static image was extracted from each clip and was then analysed with Horos™ software v2.1.1 (Horos Project, Annapolis, MD, USA). Epidermal thickness, seen as a superficial hyperechoic band, was calculated as the mean value obtained from 5 measurements at different points of the image. Grayscale levels were then analyzed by positioning circular regions of interest (ROI) in the epidermal and dermal areas. Specifically, it was calculated the mean grayscale value (MGV) within the ROI, whose values range from 0 (totally black) to 255 (total white). The expert examiner was calibrated to improve intra-examiner repeatability on ten patients not included in the study until a k value > 0.8 was obtained.

### 2.3. Statistical Analysis

Categorical data were described by absolute and relative (%) frequency, whereas continuous data by mean and standard deviation. Comparisons between mean values were analyzed by Student’s *t* test for independent samples (two-tailed) and one-way ANOVA. Comparisons between proportions were made with z-test for proportions. The correlation between variables was examined with Pearson’s correlation coefficient (r). *p* values of less than 0.05 were considered significant, and all analyses were carried out with SPSS v.22 technology (IBM Corp., Armonk, NY, USA).

## 3. Results

### 3.1. Patients and mRSS

Forty-seven SSc patients were enrolled for this study, along with 15 HC comparable for age and sex. The baseline characteristics of the SSc cohort are reported in Table 1.

Among the possible correlations with disease characteristics, the mRSS performed in the whole body was found to be significantly higher in patients with Scl70 positivity (*p* = 0.05) and hand contractures (*p* < 0.001). In contrast, lower values were associated with ACA positivity (*p* = 0.015). As expected, mRSS in dcSSc was significantly greater than lcSSc and ssSSc (*p* < 0.001 for both). Even when considering mRSS performed in the different regions of interest, dcSSc patients had significantly higher values than lcSSc and ssSSc (*p* < 0.001 for all).

### 3.2. Durometer

Durometer measurements performed in the different regions of interest highlighted that DH presented lower values in females (*p* = 0.025) and was inversely associated with disease duration (*p* = 0.012, ρ = −0.38). Among skin subsets, a statistical difference was found only at the level of IP, with dcSSc having higher values than lcSSc (*p* = 0.029) and ssSSc (*p* = 0.002). Durometer measurements were lower in ACA patients than Scl70 ones both in IP (*p* = 0.008) and PP (*p* = 0.001). Hand contractures were associated with higher values at DP (*p* = 0.009) and VF (*p* = 0.046). No other associations were found with other disease characteristics.

Evaluating possible relationships between the durometer and the total mRSS (whole body), it was found a direct correlation at the level of the IP (*p* = 0.025, ρ = 0.34), PP (*p* = 0.03, ρ = 0.33) and VF (*p* = 0.02, ρ = 0.35).

### 3.3. UHFUS

**Figure 2:**
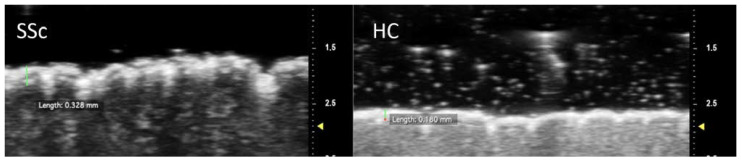
Measurement of epidermal thickness at the intermediate phalanx of the second finger. Note the increased thickness of the epidermal layer in the SSc patient compared to the control subject.

Table 2 summarizes the main UHFUS findings in our cohort. When performing UHFUS between SSc patients and HC, the former had a significantly thicker epidermal layer in all the different regions of interest (*p* < 0.001 for all)—Figure 2.

**Figure 3:**
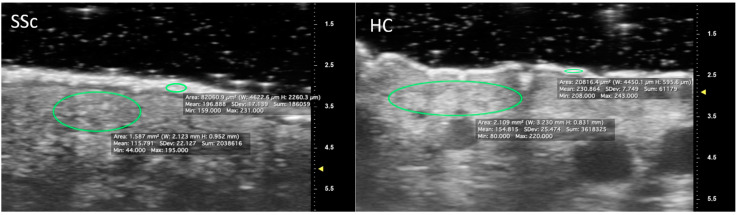
Measurement of epidermal and dermal grayscale values using ROIs positioned at the intermediate phalanx of the second finger. Note the reduction in the mean grayscale value in the SSc patient compared to the control subject in the epidermal and dermal areas.

The SSc group also presented lower epidermal MGV at DP, IP and PP (*p* = 0.01 for all). Similarly, low values of dermal MGV reached statistical significance at DP (*p* < 0.001), IP (*p* = 0.006) and PP (*p* = 0.04)—Figure 3. When UHFUS results were diversified according to skin subset, both lcSSc and dcSSc reconfirmed a significantly thicker epidermal layer than HC for all the regions of interest. Noteworthy, when considering MGV differences between cutaneous subsets and HC, statistically significant lower values were detected for both skin subsets only at the dermal layer of DP (*p* = 0.001 for lcSSc; *p* = 0.008 for dcSSc) and IP (*p* = 0.05 for lcSSc; *p* = 0.01 for dcSSc).

Among SSc patients, epidermal thickness revealed an inverse correlation with disease duration at VF (*p* = 0.017, ρ = −0.346). No associations were found between UHFUS results and skin subsets, autoantibody profile, and disease characteristics.

No relationships were found between UHFUS results, and mRSS performed in the whole body or the different regions of interest. Furthermore, when comparing outcomes obtained from UHFUS with those from durometer, no relevant associations were found for any region of interest.

## 4. Discussion

Since skin thickness is the most evident feature of SSc, several techniques have been used over the years for its evaluation, always looking for the highest validity and reliability. In our work, we assessed skin involvement in a cohort of SSc patients through mRSS, durometry and UHFUS. Furthermore, the outcomes of these techniques were compared. In addition, UHFUS results from a group of HC were also analyzed, finding significant differences between the subgroups, interesting correlations with disease characteristics, and finally, obtaining a complete multiparametric non-invasive assessment of skin involvement in SSc.

Durometer measurements suggested that higher values are likely associated with the early phase of the disease and with some characteristics such as hand contractures and diffuse cutaneous subsets. Moreover, durometry showed a good correlation with mRSS performed in the whole body and a direct trend for the different regions of interest, reiterating the findings of previous studies [6,11].

The comparison of UHFUS outcomes between SSc patients and HC pointed out that the former had a diffusely thicker epidermal layer, even in limited cutaneous forms. However, a significant dermal impairment (corresponding to a lower MGV) could be detected only in the distal part of the hand, likely reflecting the more impacting cutaneous and microvascular alterations that occur in the extremities. In this regard, an interesting negative correlation between UHFUS skin thickness and capillary density assessed by nailfold capillaroscopy was recently highlighted [12].

UHFUS showed that epidermal thickness was inversely correlated with disease duration. Hence, we confirm that skin is thicker in early phases and then becomes thinner, which was already demonstrated with HFUS by Hesselstrand et al., who found that patients with shorter disease duration had high skin thickness and low echogenicity [13]. They suggested that this ultrasonographic pattern could identify the edematous phase that precedes palpable skin involvement. This hint was somehow strengthened by an HFUS study that showed how skin thickness was more significant in SSc patients in the edematous phase and progressively decreased in those in the fibrotic and atrophic phases [14]. Moreover, a longitudinal evaluation revealed that a decrease in skin thickness and an increase in echogenicity were observed during a one-year follow-up [15].

The lack of correlations between UHFUS and both mRSS and durometry that emerged in our study is discordant with findings from other HFUS studies [13,14]. However, it could be explained by the fact that these are not equivalent techniques but complementary. The hypothesis that skin ultrasound can show something that other methods do not grasp was recently strengthened also by other studies. For example, HFUS revealed subclinical dermal involvement in areas with a normal mRSS in lcSSc patients [16], and there are other works besides ours that found no ultrasound differences between skin subsets [17]. Kissin et al., in 2006, tried to compare these three techniques for a skin assessment and found a good correlation between them all [18]. However, even leaving out the smaller number of patients analyzed, they used a 10 MHz probe, so it is reasonable to expect different and more accurate results with a 70 MHz probe. As pointed out by the Authors themselves, the durometer is designed to measure skin hardness, so it could not provide information about other pathological skin properties.

The results of this study indicate that, whereas mRSS and durometry are two techniques able to evaluate skin hardness reliably and unanimously, UHFUS appears as an autonomous method capable of showing different skin characteristics. The variation of the thickness and echogenicity of the various skin layers concerning the different phases of the disease represents an important reason for the larger use of this technique in SSc. Our data reconfirm the great potential of UHFUS in SSc skin assessment, as already highlighted by the previous work by Naredo et al. [8].

Recently, two systematic literature reviews on US assessment of skin involvement in SSc were published. They agree that the US, especially when applied with higher frequencies, is an effective and helpful tool for skin assessment in SSc, but some problems still limit its use. For example, image acquisition and analysis methods were heterogeneous and frequently under-reported, thus precluding data synthesis across studies. Moreover, there is limited or absent US evidence for sensitivity to change, test-retest reliability, clinical trial discrimination or thresholds of meaning. It was also underlined that US findings should be compared to skin histology. In this regard, although the US proved valid and reliable for skin thickness measurement, echogenicity seems to have a limited validity. Finally, developing a protocol for US skin assessment with image acquisition and analysis standardisation was deemed necessary for future research and to foster its clinical use [19,20].

Our work presents some limitations. The relatively small size of the cohort limits the generalizability of the findings to larger populations of SSc patients. Due to the intrinsic setting features of the device used for ultrasound assessment, it was impossible to measure the dermis’s thickness as the boundaries of this layer were indistinguishable from the underlying hypodermis. As pointed out by a systematic literature review as part of a large international collaborative work on the assessment of skin involvement in SSc, it should also be considered that the disease progression rate of SSc before study entry may significantly impact the results [21]. The same applies to immunosuppressive therapies that may have affected skin involvement. The study’s primary limitation is the lack of skin biopsy samples, which would have allowed us to directly validate the results obtained and the speculations formulated with the other techniques. This is even more true since a relationship between skin HFUS and dermal collagen content was found in SSc cutaneous biopsies [22]. Moreover, a good correlation between US-measured skin thickness and histological cutaneous thickness was demonstrated [23] as well as with circulating fibrocytes [24]. Skin US assessment, especially UHFUS, was performed in a few heterogeneous studies, so it still lacks validity criteria: further studies are then required. Finally, the design of the study does not provide information about the evolution of skin involvement. In this regard, a future study proposal could address UHFUS follow-up of SSc patients to assess the predictive value of UHFUS measurements in disease progression and outcomes. 

In conclusion, skin evaluation in SSc patients could benefit from complementary methods to obtain a complete assessment, especially concerning UHFUS, a relatively new technique with great potential.

## 5. Conclusions

UHFUS is an emergent non-invasive diagnostic tool for skin assessment in SSc. It showed significant alterations concerning skin thickness and echogenicity when comparing SSc patients with HC. However, the lack of correlations between UHFUS and both mRSS and durometry suggests that these are not equivalent techniques but may represent complementary methods for a full skin evaluation in SSc. Therefore, future clinical use of UHFUS in the skin evaluation of SSc patients is conceivable.

## Figures and Tables

**Table 1:** Baseline characteristics of SSc cohort and HC.

	SSc (*n* = 47)	HC (*n* = 15)	*p*
Female	41 (87.2%)	11 (73.3%)	n.s.
Age (years)	56.4 ± 13.5	54.7 ± 14.3	n.s.
Disease duration (years)	10.8 ± 10.3		
ACA	27 (57.4%)		
Scl70	16 (34%)		
- ssSSc	9 (19.1%)		
- lcSSc	27 (57.4%)		
- dcSSc	11 (23.4%)		
DUs history	22 (46.8%)		
Hand contractures	5 (10.6%)		

Data are expressed in number (percentage) or mean ± standard deviation. SSc: systemic sclerosis; HC: healthy controls; ACA: anti-centromere autoantibodies; Scl70: anti-topoisomerase I autoantibodies; ssSSc: sine scleroderma SSc; lcSSc: limited cutaneous SSc; dcSSc: diffuse cutaneous SSc; DUs: digital ulcers; n.s.: not significant.

**Table 2:** UHFUS findings in SSc and HC.

	SSc (*n* = 47)	HC (*n* = 15)	*p*
Epidermal thickness (µm)			
- DP	258.6 ± 64.2	176.5 ± 21.1	<0.001
- IP	238.4 ± 77.7	173.3 ± 20.3	<0.001
- PP	206.9 ± 42.9	156.2 ± 18.1	<0.001
- DH	182.1 ± 33.3	146.7 ± 12.7	<0.001
- VF	180.1 ± 36.4	143.0 ± 18.0	<0.001
Epidermal MGV (0–255)			
- DP	168.6 ± 40.6	191.0 ± 23.7	0.01
- IP	168.2 ± 37.8	195.6 ± 20.9	0.01
- PP	174.2 ± 38.8	193.4 ± 18.8	0.01
- DH	184.5 ± 27.7	195.1 ± 19.2	n.s.
- VF	185.4 ± 24.6	190.1 ± 39.9	n.s.
Dermal MGV (0–255)			
- DP	62.9 ± 33.5	100.8 ± 27.2	<0.001
- IP	70.0 ± 35.6	98.3 ± 25.8	0.006
- PP	87.0 ± 37.9	107.4 ± 13.8	0.04
- DH	109.4 ± 32.7	118.1 ± 22.0	n.s.
- VF	124.7 ± 33.8	130.6 ± 31.5	n.s.

Data are expressed in mean ± standard deviation. SSc: systemic sclerosis; HC: healthy controls; MGV: mean grayscale value; DP: distal phalanx; IP: intermediate phalanx; PP: proximal phalanx; DH: dorsum hand; VF: volar forearm.; n.s.: not significant.

## Data Availability

The datasets used and/or analysed during the current study are available from the corresponding author on reasonable request.
